# Dysphagia After Esophageal Replacement and Its Treatment

**DOI:** 10.1007/s00455-023-10557-2

**Published:** 2023-01-31

**Authors:** Örs Péter Horváth, Gábor Pavlovics, László Cseke, András Vereczkei, András Papp

**Affiliations:** Department of Surgery, Medical Center, Pécs University, Ifjúság u. 13, 7624 Pécs, Hungary

**Keywords:** Dysphagia, Esophageal replacement, Complications, Treatment

## Abstract

Dysphagia occurs temporarily or permanently following esophageal replacement in at least half of the cases. Swallowing disorder, in addition to severe decline in the quality of life, can lead to a deterioration of the general condition, which may lead to death if left untreated. For this reason, their early detection and treatment are a matter of importance. Between 1993 and 2012, 540 esophageal resections were performed due to malignant tumors at the Department of Surgery, Medical Center of the University of Pécs. Stomach was used for replacement in 445 cases, colon in 38 cases, and jejunum in 57 cases. The anastomosis with a stomach replacement was located to the neck in 275 cases and to the thorax in 170 cases. The colon was pulled up to the neck in each case. There were 29 cases of free jejunal replacements located to the neck and 28 cases with a Roux loop reconstruction located to the thorax. Based on the literature data and own experience, the following were found to be the causes of dysphagia in the order of frequency: anastomotic stenosis, conduit obstruction, peptic and ischemic stricture, foreign body, local recurrence, functional causes, new malignant tumor in the esophageal remnant, and malignant tumor in the organ used for replacement. Causes may overlap each other, and their treatment may be conservative or surgical. The causes of many dysphagic complications might be prevented by improving the anastomosis technique, by better preservation the blood supply of the substitute organ, by consistently applying a functional approach, and by regular follow-up.

## Introduction

The stomach, the colon, and the jejunum may all be used for esophageal replacement in the order of frequency. Typically, the following symptoms occur postoperatively as complications: dysphagia, reflux, delayed gastric emptying, early satiety, dumping syndrome, weight loss, and chronic diarrhea, which appear with different frequencies depending on the organ used for the replacement. Dysphagia occurs in approximately 50–65% after the procedures, the severity of which is classified into three categories: mild, moderate, or severe, according to the quality-of-life tests. In mild cases, solid food sometimes causes difficulty in swallowing, while in a medium grade type the pasty and in the severe cases already the liquid diet causes symptoms [1]. Swallowing disorder is the most common after gastric replacement, but complaints can decrease significantly over time [2]. The main causes of dysphagia are anastomotic stenosis, conduit obstruction, ischemic and peptic stricture, foreign body or food impaction, recurrent or new tumor in the esophagus and/or in the organ used for replacement, and functional causes. Contrast-enhanced swallowing and endoscopy are the most important tools in their investigation, but manometry, pH-metry, scintigraphy, impedance measurement, and neurologic examination methods may also be required. Endoscopic methods play a leading role in their treatment, but surgery and medication may also be considered.

## Material and Methods

In the 20 years between January 1, 1993 and December 31, 2012, 540 esophageal resections were performed due to malignant tumors at the Department of Surgery, Medical Center of the University of Pécs (Table 1.). Meanwhile further 62 esophageal resections were performed due to benign lesions, for various reasons, such as corrosive and peptic stricture, achalasia, esophageal perforation, and failed antireflux surgery. Due to the special considerations in the indication, in the planning, and implementation of operations, these were not merged into the surgical complications of the malignant tumors. However, some special aspect that may cause dysphagia, which were not detected after operations for benign diseases are also highlighted.

**Table 1:** Demographic data

Age (years)	60(41–69)
Female:male ratio	1:8,1
Tumor location	
Pharyngo-esophageal junction	29
Upper third	147
Middle third	245
Lower third	119
Histology	
Squamous cell cancer	439
Adenocarcinoma	101
Neoadjuvant treatment	119

The first option for a replacement was the stomach, due to its simplicity and good blood supply. If the stomach was not suitable, the large intestine was chosen. Small intestine was considered only after the resection of lower third tumors or as a free transplant.

In case of a gastric replacement, the stomach was prepared according to Akiyama through an abdominal approach combined with a suprapancreatic lymph node dissection [3]. Afterward either a transmediastinal esophagectomy and a handsewn anastomosis on the neck was performed or from a right posterolateral thoracotomy a subtotal esophagectomy with stapled anastomosis was created.

The large intestine was chosen for a substitute if the stomach was not suitable for replacement. The proper part of the colon was chosen from an abdominal exploration. The first option was always the left colon; however, if the blood supply was not reliable for anatomical reasons, the right half of the colon was applied. The esophagus was removed without a thoracotomy and the colon segment was pulled up to the neck in the posterior mediastinum. On the neck a handsewn anastomosis was created [3].

Jejunum was used for a replacement in two modalities [3]. In case of a tumor located to the pharyngo-esophageal junction, a pharyngo-laryngectomy was performed with the removal of the neck section of the esophagus, and the replacement was performed with a free jejunal transfer. For tumors located in the lower third, the esophagus was resected through a left thoracolaparotomy and the stomach was totally or subtotally removed. The replacement was performed with a straight Roux loop with a handsewn anastomosis,

In case of an advanced stage cancer with uncertain resectability without distant metastases, a preoperative oncologic treatment was routinely performed. In 2010, we reported 73 successful neoadjuvant treatments for squamous cell tumors and did not detect any difference in the anastomosis complications compared to the control group without treatment [4]. In the examined 20-year period, there was only one significant change in the surgical technique, namely the introduction of the laparoscopic technique. In the last 5 years, in 20 selected cases, this type of surgical intervention was performed without any change in the oncological principles or in the replacement strategy of the esophagus (mechanical esophago-gastrostomy performed in the chest). No difference in the complications were observed after the laparoscopic operations.

We have reliable data on dysphagic complications only for the first two months after surgery, because about a third of our patients did not appear for the later controls for complex reasons (social status, social-security rules, compliance, and possibly no complaints) and the 5-year survival is low 15% [4]. The distribution according to the replacement method and the dysphagic complications occurring in the first two months are shown in Table 2.

**Table 2:** Patients with esophageal resections according to the organs used for replacement and anastomosis complications

Organs used for replacement		Number	Conduit necrosis	Anastomotic insufficiency	Early anastomotic stenosis within 1 year
Stomach	cervical anastomosis	275	6 (2.2%)	49 (17.8%)	15 (5.6%)
	thoracic anastomosis	170	2 (1.2%)	6 (3.5%)	0
Colon		38	3 (8%)	2 (5%)	0
Jejunum	Roux loop with thoracic anastomosis	28	0	1 (3%)	0
	free jejunal transfer to the neck	29	1 (3%)	0	1 (3%)

## Discussion

Dysphagia following esophageal resection and replacement may seriously affect the quality of life, lead to malnutrition due to the eating disorder, and a consequential aspiration may result in pulmonary complications and death of the debilitated patient following a treatment failure. In anatomical order, dysphagia may develop in the oropharynx (motility disorder, high pharyngeal anastomosis), in the residual esophagus (stricture, primary or recurrent tumor), in the anastomotic region (technical reason, ischemic, and/or peptic stenosis), in the organ used for replacement (motility disorder, stricture, tumor), or at the hiatus or pylorus. Patients coming for follow-up visits are asked to fill in a questionnaire, to determine the degree of dysphagia, and to evaluate how the quality of life is affected by his complaints. As the first examination, a contrast swallow is performed, which directs further examinations. If a stricture is detected, the mandatory next examination is endoscopy to rule out recurrent tumor and estimate the degree of stricture and possibly its etiology (e.g., whether the area around the anastomosis is covered with bile or is inflamed due to acid reflux). In suspicious cases (Barrett’s esophagus), a biopsy for histology is taken. In case of reflux symptoms and endoscopic signs, a 24-h pH-metry, and in the case of motility disorders, a manometry is indicated. The causes of swallowing difficulties and the options for prevention and treatment are described in order of frequency, as follows.

### Anastomotic Stenosis

**Fig. 1:**
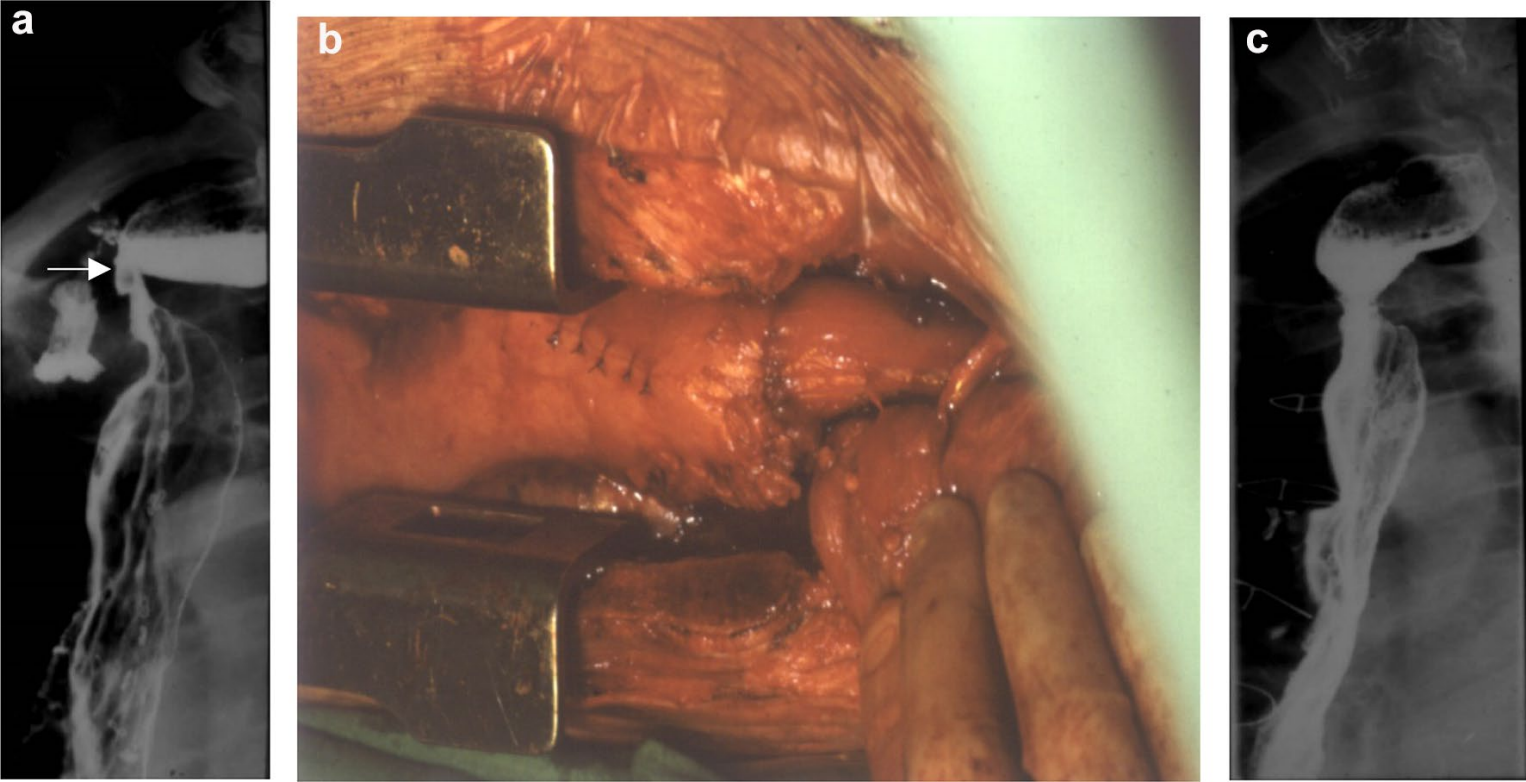
**a** Anastomotic stenosis with insufficiency (arrow) after substernal gastric replacement. **b** The re-sutured esophago-gastrostomy from median sternotomy. **c** Contrast swallow showing free passage

The exact definition of anastomotic stenosis is difficult to describe. If a patient has dysphagia and the diagnostic endoscope is not able to pass through the stenosis (< 13 mm), it is already a stricture requiring therapy [5]. It is known that even with radiologically and endoscopically detected stenosis, a patient does not always have a complaint which is called as an asymptomatic stricture [6]. However, a less severe stenosis can also cause a difficulty in swallowing due to the presence of a recurrent laryngeal nerve palsy or an oropharyngeal motility disorder [7]. The incidence of the anastomotic stenosis depends on several factors. Most commonly this occurs after a gastric pull-up and the incidence of a stenosis within one year can be as high as 50–85% [8–10]. After colon and small bowel replacements, its frequency is below 10% [11, 12]. In our patient material, anastomotic strictures occurred in 5.6% of cervical anastomoses sutured with the stomach, while strictures never developed in those sutured with the colon or jejunum. Mortality is significantly lower in benign cases (1% vs 7%), so the complication rate also corresponds to this (3) because, in benign cases, only colon and jejunum were used for replacement, where anastomotic complications are significantly lower. The most common cause of dysphagia is a healed anastomotic insufficiency, although observations suggest that postoperative strictures are twice as common as insufficiencies. A stricture which develops after more than a year is always suspicious of a recurrence. Further causes may include ischemia [10], radiotherapy [13], biliary or acidic reflux, and angulation following substernal or antethoracic replacements. It is less common in an anastomosis located to the chest compared to those made in the neck [14]. The anastomotic insufficiency in the neck was four times more frequent than in the chest in our material (17.8% vs 3.5%). Zhu et al. [15] found that an anastomotic stricture is significantly more frequent if the gastric tube is wider than 5 cm or the whole stomach has been used for replacement. The diameter of a circular stapler or a technical failure may also play a role. The strictures are more common with the OrVil technique compared to the conventional circular stapler [12, 16]. According to Petrin et al. [17], the stenosis rate was 62.5% with a 21-mm anvil, 16.7% with a 25-mm anvil, and only 5.1% with a 28-mm anvil. The best results can be obtained by a side-to-side anastomosis created with an endoGIA stapler, although this technique cannot always be applied in the neck or high in the chest [18]. The vast majority of strictures can be treated with dilation, laser coagulation, or stenting [9]. In cases, which are difficult to be dilated, an internal radial incision of the stricture [19] or even an endoscopic circular excision [20] may be used. Steroid infiltration of the stenosis has been reported by several authors to improve the effect of an endoscopic dilatation [19, 21]. Very rarely, plastic surgery (strictureplasty, applying musculocutaneous flaps) or reoperation from a median sternotomy can be the solution [22]. In one of our cases after a substernal gastric pull-up, a 90-degree angulation and stricture developed at the level of the anastomosis which could not be dilated. From a median sternotomy the stomach was mobilized and a new esophago-gastrostomy was created. The patient’s swallowing difficulty resolved afterward (Fig. 1a–c.).

### Conduit Obstruction

**Fig. 2:**
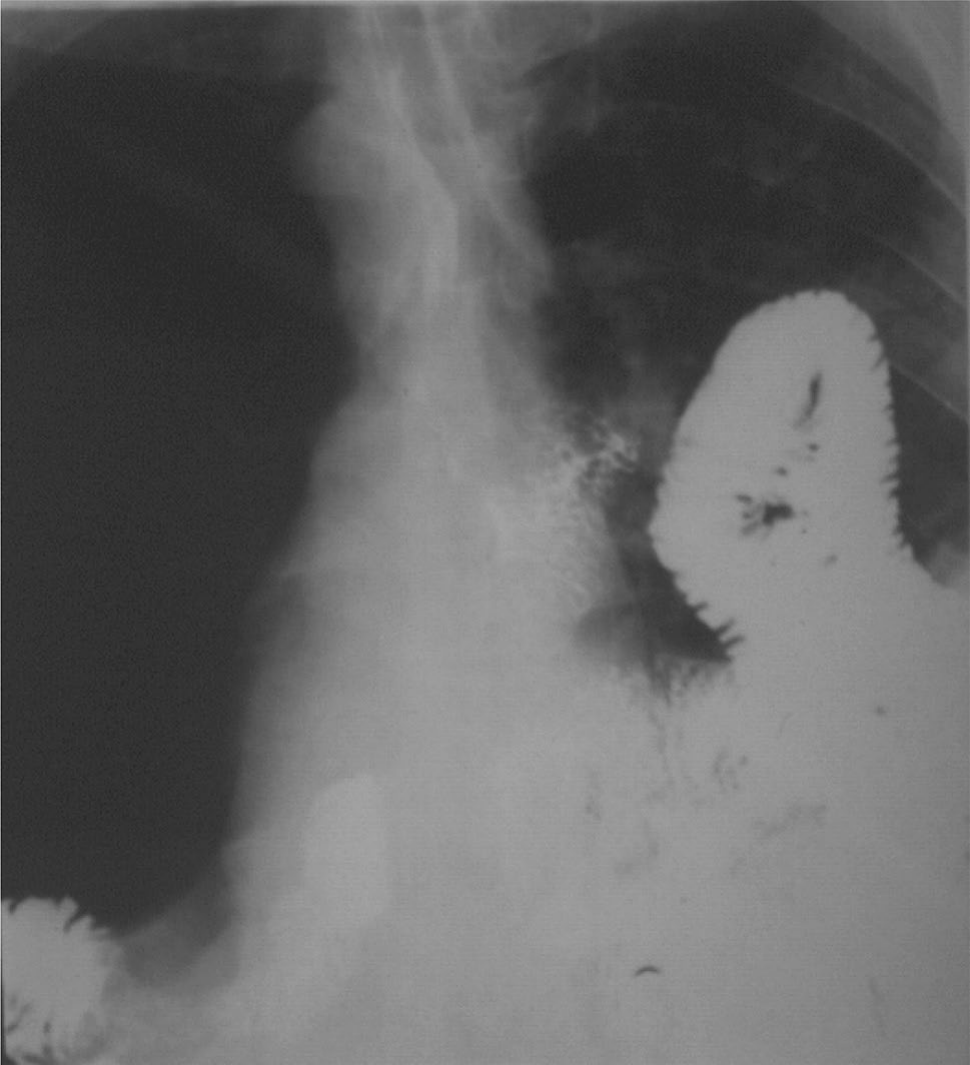
Jejunal loop herniation in the chest after a cardia resection

At the time when the surgical treatment of an ulcer disease was general, it was a textbook rule that a drainage procedure (pylorus plasty, GEA) is mandatory in addition to the truncal vagotomy due to a pyloric stenosis occurring in almost 50% of cases. This rule has been adapted to esophageal resections and gastric replacements because the procedure comprises a truncal vagotomy. In case of an intact pylorus, there was a fear of an increase in the number of anastomotic insufficiencies and an increase in pulmonary complications due to the troubled gastric emptying [23]. Observations reporting later that biliary reflux can cause severe complaints and play a role in the development of late anastomotic strictures [24], most of the teams quitted the pyloroplasty and the rare, severe pyloric dysfunction could be resolved by dilation [25] or peroral endoscopic myotomy [26]. Gastric motility can also be triggered by the administration of erythromycin [25]. As a preventive intervention, the intraoperative injection of botulinum toxin into the pylorus has been introduced, which is also an option postoperatively by an endoscopic application [23], although conflicting result have been reported with this method [27, 28]. In our practice, we used the so-called finger fracture method initially, then abandoned it later completely, and have not seen any gastric emptying disturbances. After gastric replacement, delayed gastric emptying develops in about 10–20% [29], which can be caused by a non-relaxing pylorus [30], an impaired gastric peristalsis or due to the unfavorable pressure conditions as the pressure in the chest is negative, while in the abdominal cavity it is positive. Other causes may include torsion or angulation of the pulled up stomach, a redundant conduit, and a tight hiatus. In the early postoperative period, gastric emptying is impaired by the paralytic ileus of the intestines, as well as by the presence of anastomotic and pulmonary complications. Complications can be reduced by early mobilization and early oral feeding. Gastric peristalsis may also improve spontaneously over time [27]. In his study, Arya [31] found that abandoning pyloroplasty does not increase either the number of anastomotic insufficiencies or pulmonary complications. We present a case of a conduit obstruction after a cardia resection with jejunal replacement, when two jejunal loops slipped into the thoracic cavity and caused a difficulty in swallowing (Fig. 2). A reoperation was performed from an abdominal approach, the small intestine was retracted, and the wide hiatus was narrowed. Our case also shows that such a complication can be prevented by properly narrowing the hiatus at the primary operation.

### Peptic Stricture

Peptic stricture occurs mostly after gastric replacements. An anastomosis can be created high in the thorax or in the neck with the same functional consequences; however, a lower position is more likely to be associated with a gastroesophageal reflux. Reflux may be particularly pronounced in an anastomosis performed below the bifurcation. Reflux can be reduced by making the stomach tube very narrow and abandoning the pyloroplasty. The dilatation of a stricture caused by the biliary reflux was more difficult in our experience and the interval between dilations could not be increased. While a stenosis without a reflux usually heals after two dilations, those caused by biliary reflux must be dilated for even years [30]. Acidic and mixed reflux may lead to columnar metaplasia above the esophago-gastric anastomosis, in up to 50% of the cases according to some authors and in about 30% an esophageal stricture will also develop [32–34]. Gutschow et al. [35] described the development of an adenocarcinoma in the remnant esophagus 28 months after an esophageal resection and gastric replacement. For the prevention of strictures caused by reflux some authors recommend a recessed anastomosis [36] others a postoperative PPI treatment [37] or prokinetic drugs [38]. Complete prevention of the reflux is not attainable even in a 15–20-cm-long jejunal segment, proven by one of our cases, where an esophageal adenocarcinoma developed above a jejunal interposition 18 years after an esophageal resection for a benign cause [39]. There may be overlaps between peptic strictures and ischemic anastomotic stenosis that can be differentiated according to the length of the stenosis, as peptic strictures are usually longer.

### Ischemic Stenosis

The blood supply of the esophagus is reliable which permits the preparation of a 4–5-cm-long segment without any problem in its perfusion. If a patient must be reoperated due to a conduit necrosis, the circulatory disorder is always found in the organ used for the replacement and never in the esophagus. Three groups are distinguished according to the degree of the ischemic damage [40]. In the first, the circulatory disturbance is limited to the mucosa, endoscopy shows a dark bluish lesion, no surgical intervention, and only endoscopic controls are required to monitor the progress of the process. In the second, focal necrosis appears and clinically an anastomotic insufficiency is observed. Interventional endoscopy (clipping, negative pressure therapy, stent implantation), possibly drainage, and later surgical correction may be required. In the third an advanced conduit necrosis is seen; the anastomosis should be disassembled with the resection of the conduit and the construction of an esophagostoma. Reconstruction must be planned for later if the patient survives the severe septic complication. Dysphagia can be an early sign in all three stages. Grades 1 and 2 are likely to lead to an anastomotic stenosis later on. Ischemic damage occurs in 2–10% of the cases, mostly after colonic replacement 11]. The frequency of a conduit necrosis was the following in our material: colon 8%, stomach 1.8%, and jejunum (Roux loop) 0%. The graft loss after free jejunal transfer was 3%.

Risk factors for an ischemic injury can be divided into three groups [40]: 1. Risk factors of the patient include peripheral arterial vascular disease, stenosis of the coeliac trunk, heart failure, and diabetes mellitus. 2. Technical defects can occur such as conduit twisting, tight hiatus, greater-curvature arcade injury, hematoma formation, narrow chest inlet, and too narrow stomach conduit. 3. Persistent hypotension may be a risk factor in the postoperative period, especially if it is treated with vasopressors. Inhibition of the venous outflow occurs earlier, which can be detected in the form of a bluish discoloration of the graft (so-called blue loop syndrome). Endoscopic and laser Doppler monitoring may be recommended to prevent complications [40]. Indocyanine green fluorescence imaging is an emerging technology that might help in decreasing anastomotic leakage rates [41]. There is a large literature on ischemic preconditioning; however, according to meta-analyses, only severe complications can be decreased with this method [42]. The use of the supercharging technique is a very safe preventive option, especially in the case of colonic replacements [43, 44]. We provided such a supplemental blood supply for colonic replacements in eight cases and no anastomotic complications occurred. Supplementary blood supply may be provided from the neck arteries or from the internal thoracic artery. Thoracic epidural anesthesia (Th 6-10) improves the blood supply on the tip of the gastric tube based on experimental and clinical studies [45, 46] and this may reduce the risk of an anastomotic insufficiency.

**Fig. 3:**
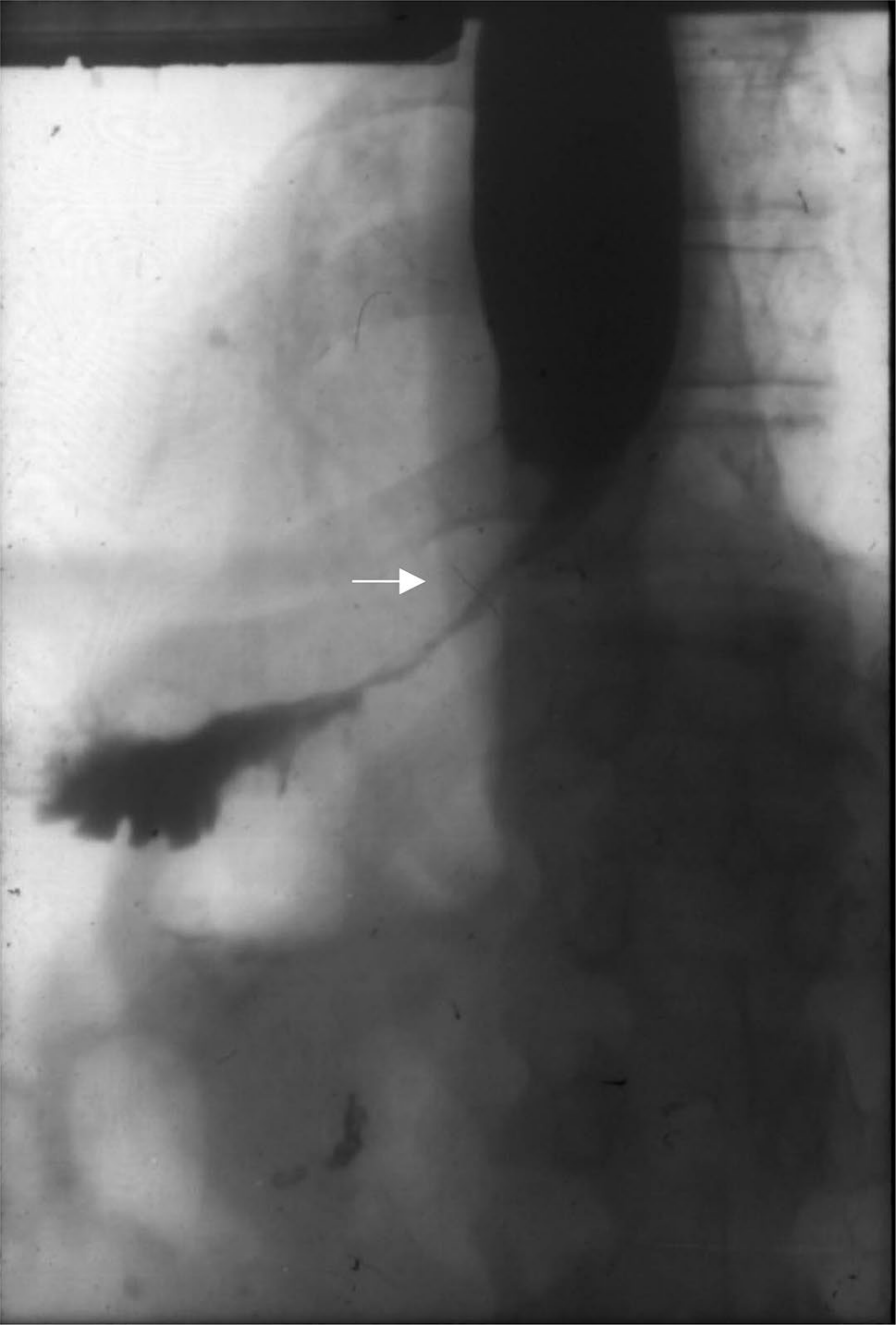
Ischemic jejunal stricture (arrow) after total gastrectomy

**Fig. 4:**
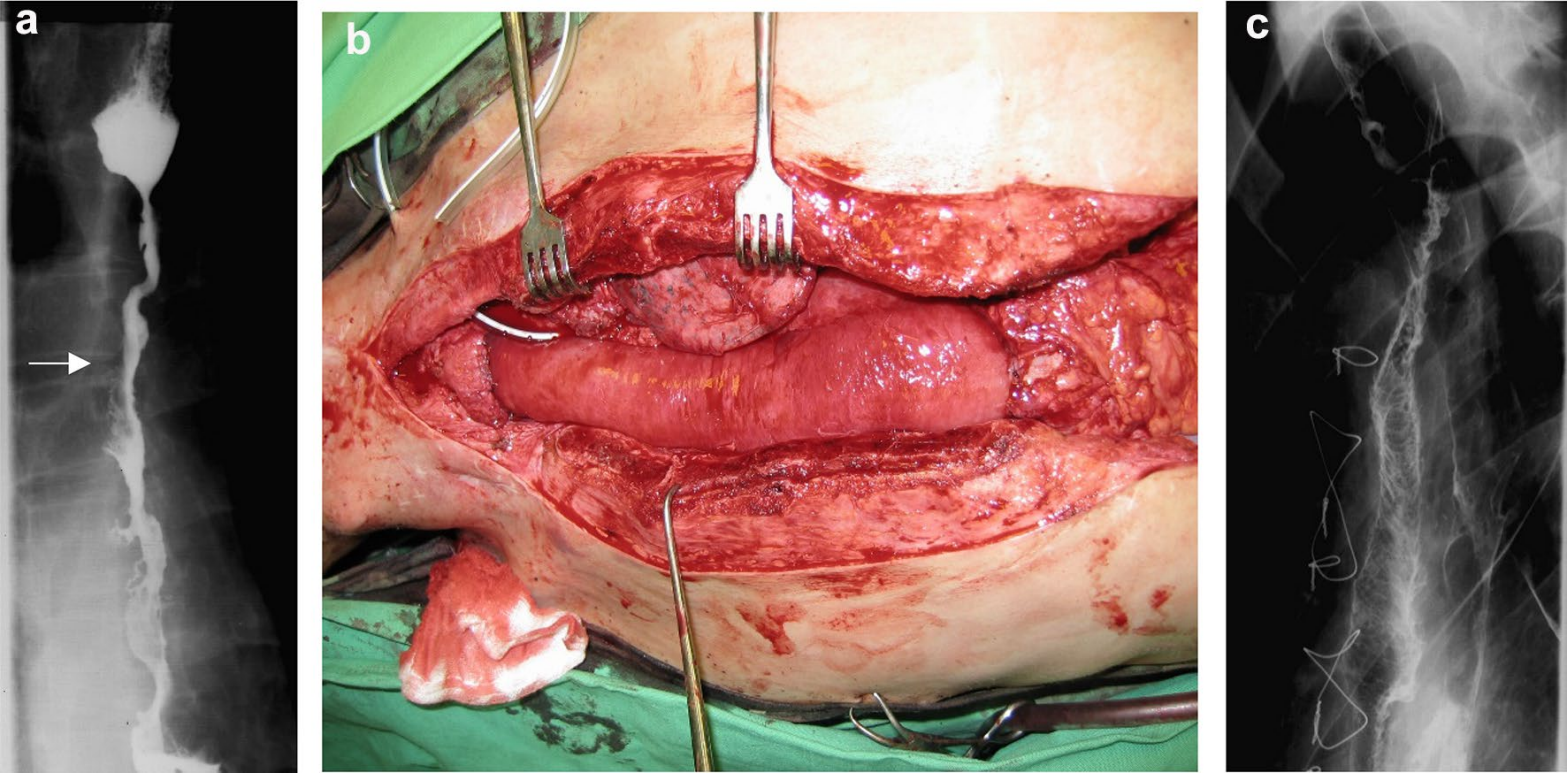
**a** Ischemic stricture (arrow) after a substernal ileo-colon replacement. **b** Free jejunal transfer from median sternotomy. **c** Substernal esophago-jejuno-colostomy

In one case, after total gastrectomy with the resection of the abdominal esophagus, we observed a long stenosis in the jejunal Roux loop (Fig. 3), which had to be replaced with a new loop after half a year [47]. In another case, a long stenosis developed after a substernal ileocolonic replacement (Fig. 4) that could not be treated with dilation, thus it had to be removed from a median sternotomy and reconstructed with a free jejunal loop. The jejunal segment received its blood supply from internal thoracic artery and the venous drainage was secured to a neck vein with a saphenous graft [48].

### Foreign Body

A foreign body obstruction of the anastomosis is the main cause of the sudden inability to swallow, after an esophageal replacement. The patient usually also indicates that the symptoms arose during eating. A contrast swallow can easily clarify the cause and an endoscopic removal is mostly successful. After foreign body removal, dilation of the anastomosis or temporary stenting may be necessary to prevent repeated food obstruction and possible choking from aspiration. Food impaction can develop in a redundant, more fold-twisted colon over the years, which sometime pose and indication for surgery [49].

### Local Recurrence

**Fig. 5:**
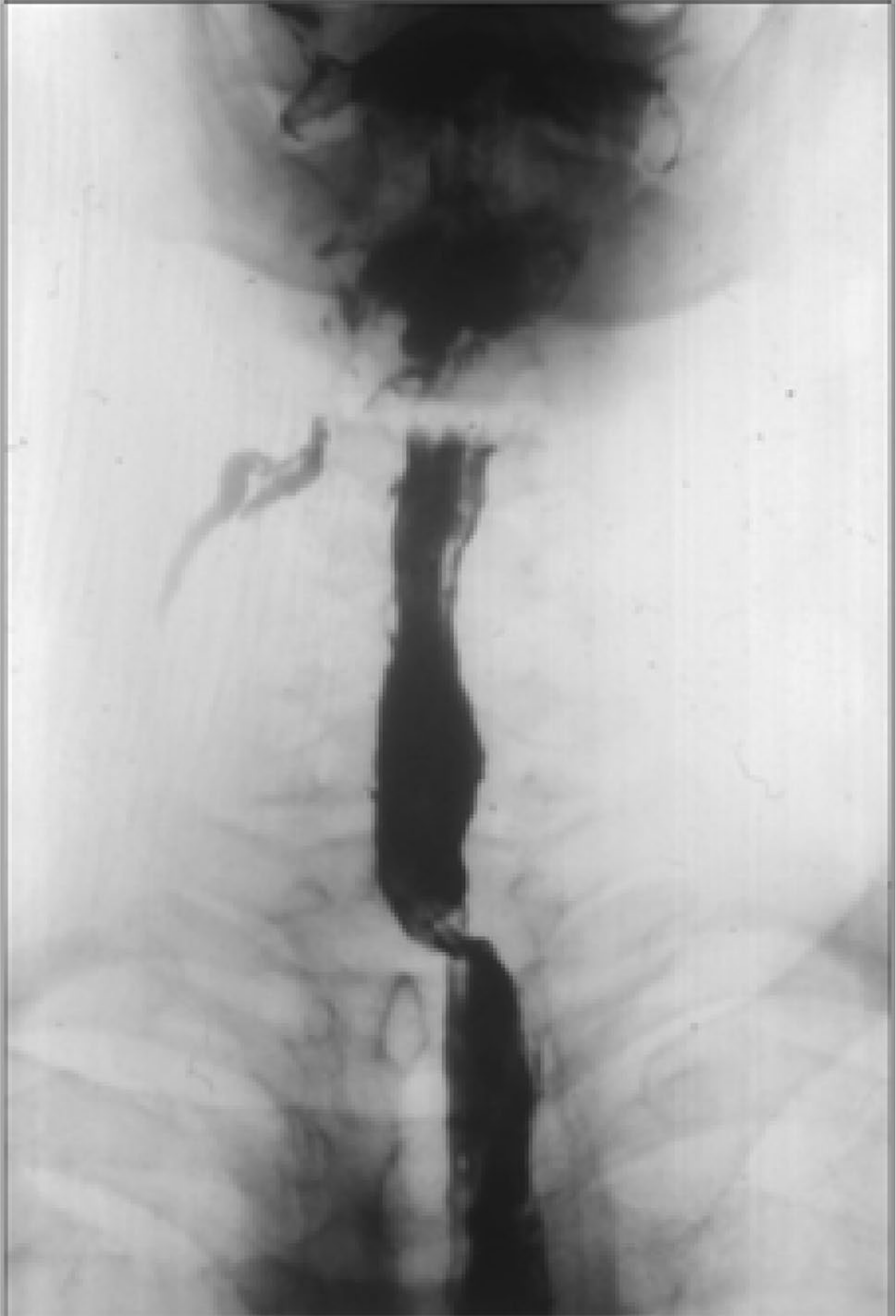
Free jejunal graft transplantation for a recurrence in the pharyngo-gastric anastomosis, after pharyngo-laryngo-esophagectomy

If the dysphagia occurs more than one year after an esophageal resection due to a malignancy, the first task is to rule out a local recurrence. Dysphagia can be caused by an intra- or extraluminal recurrence as well. The cause of a recurrence is mostly the insufficiently long tumor-free margin, which should be 2 cms for T2 and 3 cms for T3 cancers. This rule has been modified after an induction oncologic treatment for the responders, when an R0 resection itself is enough. Therefore a larynx-preserving esophagectomy can be performed in cases of pharyngo-esophageal cancers. Stent implantation or oncologic treatment is usually considered in the case of an external recurrence. However, there is a slight chance of surgical treatment if the recurrence occurs within the lumen. In one of our cases, a pharyngo-laryngectomy was performed due to an anastomotic recurrence after a previous larynx-preserving pharyngo-esophagectomy. The reconstruction was performed using free jejunal transplantation (Fig. 5).

### Functional Reasons

**Fig. 6:**
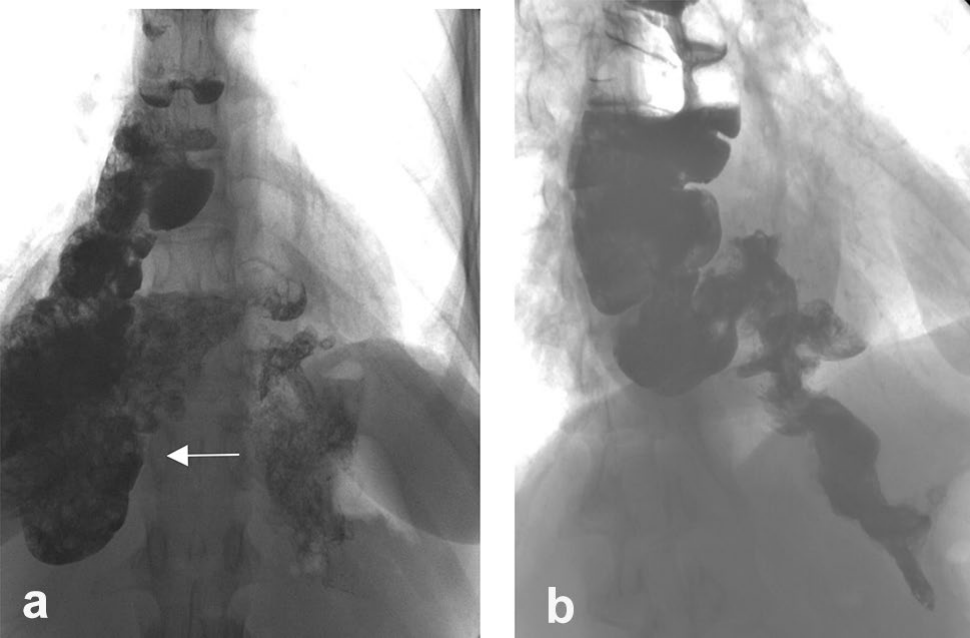
**a** Redundant colonic replacement (arrow) in the posterior mediastinum. **b** After longitudinal plication, the passage is free

**Fig. 7:**
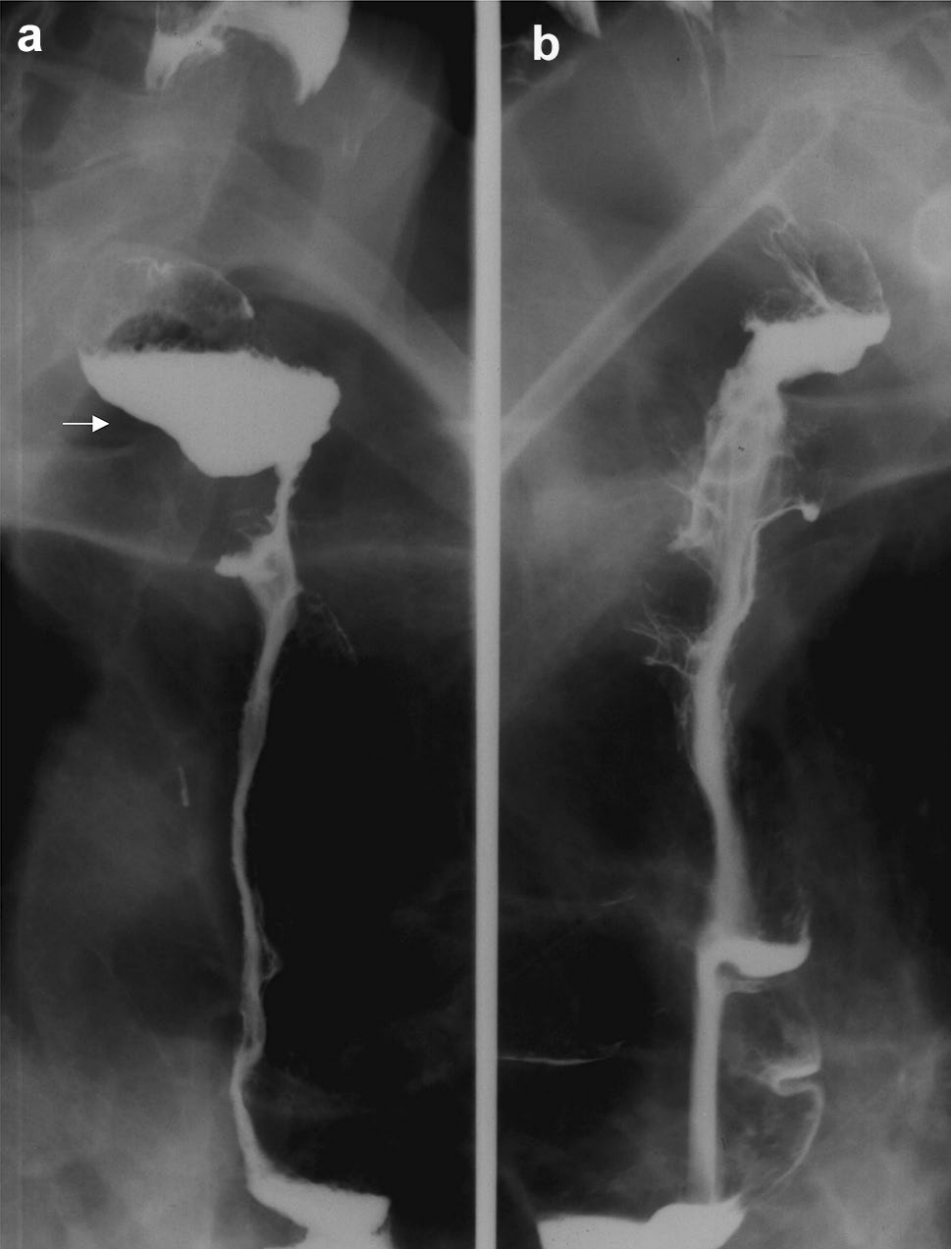
**a** Diverticulum (arrow) in the pulled up colon in the neck**. b** Contrast swallow after the plication of the diverticulum

Patients often complain that the first bites in the morning are difficult to swallow following a replacement with jejunum because the foamy saliva collects in their esophagus at night. Swallowing problems disappear some minutes later after drinking liquid. Roux stasis syndrome occurs in about 8% of total gastrectomies and may be associated with severe digestive and eating disorders, probably due to the vagotomy, causing temporary or permanent motility disorders [50]. It may also be due to the so-called Petersen hernia, when the jejunum slips behind the mesentery of the Roux loop. In most cases, the width of the anastomosis is normal. Swallowing disorders are more common with an anisoperastaltic colonic replacement; thus, this is only recommended in exceptional cases. A known late complication of an esophageal replacement with the colon is the redundancy, which can be resolved by multiple bypasses or a resection [22, 49]. In one of our cases a colonic replacement was performed in the posterior mediastinum after a transhiatal esophageal resection. Reoperation due to dysphagia was performed from a right thoracotomy, and swallowing was restored by a resection and longitudinal plication of the substitute (Fig. 6a, b). Another known complication of substernal colonic replacement is the formation of a diverticulum in the neck due to the narrowness of the thoracic inlet. In this situation, the patient tries to empty the palpable diverticulum manually, which has grown to the size of an apple on the neck, causing an esthetic problem as well. In one of our cases, the swallowing disorder has been resolved after the resection of the diverticulum (Fig. 7a, b).

### Malignant Tumor of the Esophageal Remnant

**Fig. 8:**
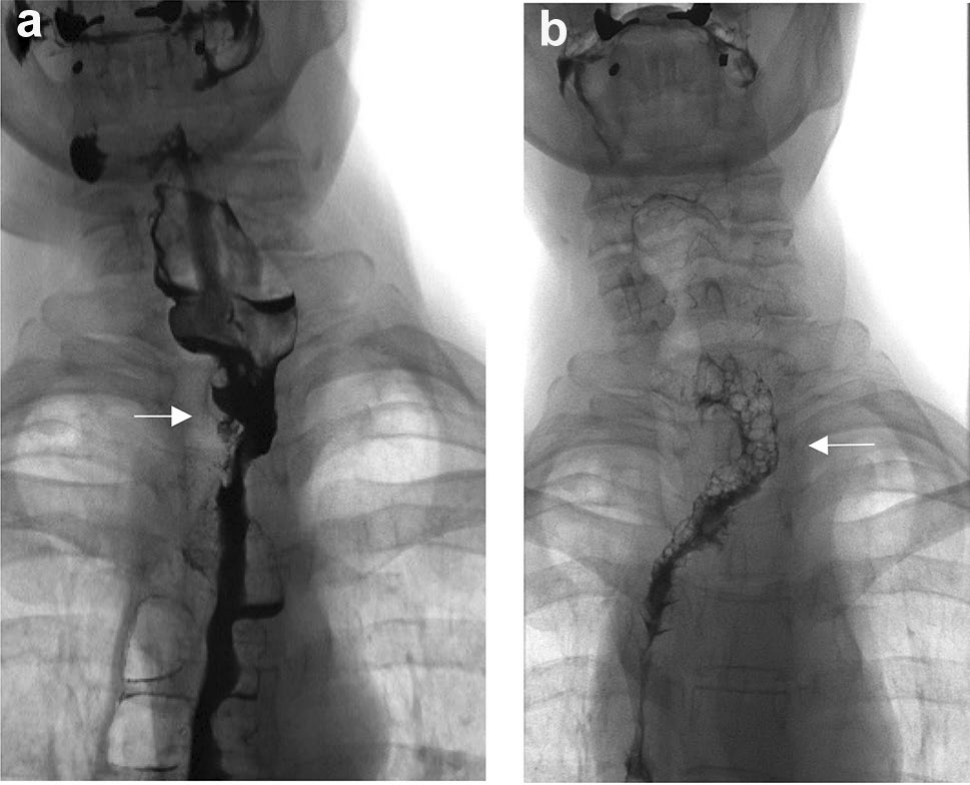
**a** Scar cancer (arrow) in the remnant esophagus after subtotal esophagectomy and intrathoracal colonic replacement performed 25 years earlier due to a corrosive stricture and **b** after pharyngo-esophageal resection and free jejunal graft interposition (arrow), contrast swallow shows pharyngo-jejuno-colostomy

If the entire esophagus is not removed during the first esophageal resection, a malignant tumor may develop in the remnant. Two such cases—a recurrent Barrett after esophago-gastrostomy causing an adenocarcinoma [35] and an adenocarcinoma developing after the resection of a benign esophageal stricture [39]—are reported in the literature. In our case, 25 years after subtotal resection of a corrosive esophageal stricture and colonic replacement, a squamous cell carcinoma (scar cancer) developed in the remnant. This could be resected after a neoadjuvant treatment and successfully reconstructed by a free jejunal transfer (Fig. 8a, b). A new cancer in the esophagus usually develops after many years or decades causing worsening dysphagia. However, the risk of developing a new tumor is considered to be low, thus the need for regular endoscopic control is questionable.

### Malignant Tumor in the Substitute Organ

**Fig. 9:**
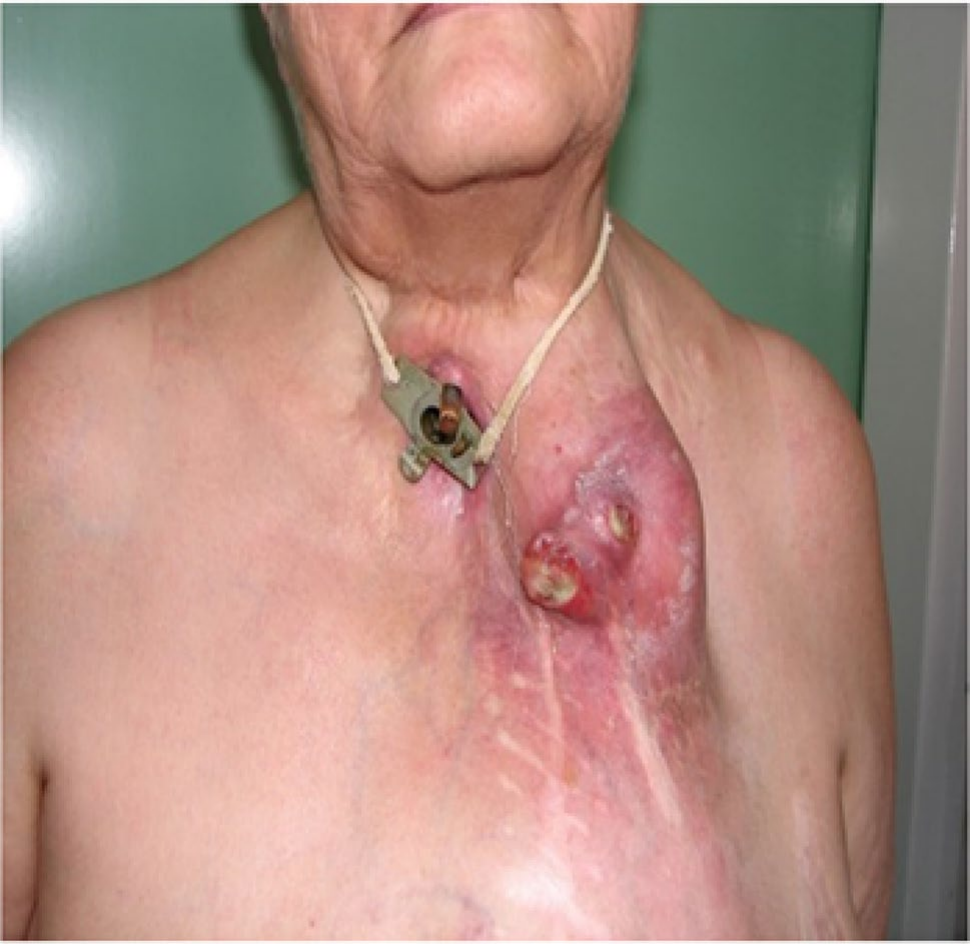
Tumor invading the skin in the antethoracal colonic substitute

The initial historic esophageal replacement method was the construction of a skin tube. Tumors in the skin tube appeared 25–30 years later, apparently caused by gastric acid and the chronic irritating effect of the diet [51]. Asian authors also described cancers of the stomach used for esophageal replacement, which is not surprising given the otherwise high incidence of gastric cancer in Asian countries [52]. Similarly, it is also not surprising that cancers have been found in the colon used for esophageal replacement. In one of our cases, a tumor developed in an antethoracic colon bypass which had been performed 36 years earlier. The colon segment was resected, and the continuity was restored by free jejunal transplantation (Fig. 9). A possible conclusion is that it may be worth performing not only an angiography before colonic replacement, but also colonoscopy with the removal of any polyps. The development of a tumor in the jejunum used for replacement has not yet been described.

Table 3 shows the frequency and treatment of complications.

**Table 3:** Prevalence and management of complications

Complications (540 patients)	Number of complications	Treatment
Anastomotic insuff	57	Endoscopy: 48 Surgery: 9
Conduit necrosis	11	Surgery: 11
Anastomotic stricture (peptic, ischemic)	15	Endoscopy (stent, drug): 15
Conduit obstruction	15	Pyloroplasty: 2 Pyloric dilation: 2 Drug treatment: 11
Foreign body	25	Endoscopy
Functional disorders	6	Surgery: 2 Drug treatment: 4
Malignant tumor in remnant or in replaced organ	5	Surgery

As a summary, the development of dysphagia is a common complication after esophageal replacements. Most of the complications might be prevented by improving the anastomosis technique, by preserving the blood supply of the organ used for replacement, and by consistently applying a functional approach. Of course, there are unavoidable causes and unforeseen complications. Resolving these requires extensive surgical expertise, combined with thorough preoperative examinations, careful postoperative surveillance and follow-up.
